# The Role of *TRAF4* and *B3GAT1* Gene Expression in the Food Hypersensitivity and Insect Venom Allergy in Mastocytosis

**DOI:** 10.1007/s00005-016-0397-7

**Published:** 2016-04-16

**Authors:** Aleksandra Górska, Marta Gruchała-Niedoszytko, Marek Niedoszytko, Agnieszka Maciejewska, Marta Chełmińska, Marcin Skrzypski, Bartosz Wasąg, Małgorzata Kaczkan, Magdalena Lange, Bogusław Nedoszytko, Ryszard Pawłowski, Sylwia Małgorzewicz, Ewa Jassem

**Affiliations:** 1 Department of Allergology, Medical University of Gdansk, Gdańsk, Poland; 2 Department of Clinical Nutrition, Medical University of Gdansk, Marii Skłodowskiej-Curie 3a, 80-210 Gdańsk, Poland; 3 Department of Forensic Medicine, Medical University of Gdansk, Gdańsk, Poland; 4 Department of Oncology, Medical University of Gdansk, Gdańsk, Poland; 5 Department of Genetics, Medical University of Gdansk, Gdańsk, Poland; 6 Department of Dermatology, Medical University of Gdansk, Gdańsk, Poland

**Keywords:** Mastocytosis, Food allergy, Food hypersensitivity, Drug hypersensitivity, Insect sting anaphylaxis

## Abstract

Mastocytosis is an uncommon disease classified as a myeloproliferative neoplasm, however, its symptoms are broad and place patients at crossroads between dermatology, hematology and allergology. Patients with mastocytosis often suffer from symptoms resulting from the activation and release of mediators from the mast cells, such as generalized itching, redness, headache, abdominal cramps, diarrhea, bone pain or arthritis, hypotension and shock. The possible severe, fatal or near fatal reactions caused by food hypersensitivity are reasons for the research focused on marker identification. The aim of the study was to analyse the gene expression differences in mastocytosis patients with and without food and drug hypersensitivity and insect venom allergy (IVA). A total of 57 Caucasian patients with mastocytosis were studied [median age 41.8; range 18–77 years; 15 (26.3 %) males and 42 (73.7 %) females]. Quantitative RT-PCRs of 11 genes plus ribosomal 18S RNA were run. Symptoms of food hypersensitivity were found in 12 patients (21 %), including 3 patients (13 %) with cutaneous mastocytosis (CM), and 9 (28 %) with indolent systemic mastocytosis (ISM). IVA was confirmed in 13 patients (22.8 %) including 6 patients (10.5 %) with CM, and 7 patients (12.3 %) with ISM. Drug hypersensitivity was diagnosed in 10 patients (17.5 %). Significant differences in the gene expression were found for TRAF4 (*p* = 0.008) in the comparison of the mastocytosis patients with and without concomitant food hypersensitivity. Furthermore significant differences were found in gene expression for B3GAT1 (*p* = 0.003) in patients with IVA compared to patients without insect sting anaphylaxis in the medical history. The expression of studied genes did not differ according to the presence of drug hypersensitivity. The TRAF4 expression was higher in mastocytosis patients with food hypersensitivity in their medical history, the B3GAT1 expression was lower in mastocytosis patients with IVA in history.

## Introduction

Mastocytosis is an uncommon disease classified as a myeloproliferative neoplasm, however, its symptoms are broad and place patients at crossroads between dermatology, hematology and allergology (Valent et al. 2007, 2012). Dutch data suggest the increasing prevalence of the disease which may be related to greater awareness of the physicians, and tryptase measurements in patients with anaphylaxis, however, the influence of environmental factors may not be excluded (van Doormaal et al. 2013). The severity of symptoms related to the mast cell bone marrow infiltration is mild in the majority of subjects classified as cutaneous or indolent. The anaphylactic reactions prevalent in approximately half of the patients constitute principal causes of the quality of life impairment and systemic life threatening reactions (Brockow et al. 2008; Jennings et al. 2014). The most important eliciting agents in mastocytosis are insect venom, food and drug allergens, and physical factors (Brockow et al. 2008; Cifuentes et al. 2013; Górska et al. 2015). Insect sting is considered a major cause of mast cell activation in patients with mastocytosis. It is estimated that 30 % of patients with mastocytosis have insect sting anaphylactic reactions (Brockow et al. 2008), which are more frequent and more severe than in general population with insect venom allergies (IVA; 1–3 %) (Ludolph-Hauser et al. 2001). Furthermore, the more severe anaphylaxis may be the effect of the activation of a cascade of intracellular tyrosine kinases: Kit, Lyn, Syk and Fyn in abnormal mast cells. However, the presence of *KIT* gene mutations, notably D816V, detectable in more than 90 % of patients with systemic mastocytosis resulting in an increased activation of mast cells, does not correlate with the severity or the prevalence of anaphylaxis (Peavy and Metcalfe 2008). The food hypersensitivity is responsible for a variety of reactions ranging from common abdominal symptoms to anaphylactic reactions. The definition of the European Academy of Allergy and Clinical Immunology describes food hypersensitivity as an abnormally strong response to a food stimulus, whereas a subgroup of immunologically mediated reactions is referred to as a food allergy (Ring 2014). Epidemiological data indicate that symptoms of food hypersensitivity are present in 17 % of the general population. A thorough diagnosis using a double blind placebo control food challenge confirmed a food allergy diagnosis in 0.9 % of the general population (approx. 5 % of patients declaring food hypersensitivity) (Nwaru et al. 2014). The Epidemiology of Allergic Diseases study (in Poland) found the symptoms of food hypersensitivity in 13 % of children aged 6–7 years, 11 % of teenagers 13–14 years and 5 % in adults (Samolinski 2015). The data of the Polish Centre of the European Competence Network on Mastocytosis indicate that symptoms related to food hypersensitivity were found in 29 % of mastocytosis patients (Górska et al. 2015). A significant infiltration of the mast cells may be found in various chronic inflammatory diseases (Henderson et al. 2012). The higher number of mast cells found in mastocytosis patients is also a risk factor to the symptoms caused by food rich in histamine, biogenic amines and histamine-releasing type of food (Vlieg-Boerstra et al. 2005). The possible severe, fatal or near fatal reactions caused by food hypersensitivity are reasons for the research focused on the identification of the markers which could be used to assess the risk and initiate the treatment to lower the severity of a possible reaction. Currently, several clinical markers can be used as the tryptase level, severity of skin involvement, and the prevalence of indolent systemic mastocytosis (ISM) (Brockow et al. 2008; Górska et al. 2015). However, the diagnosis based on the gene expression, common method used in oncology and hematology, may improve the diagnosis and tailor the therapy. Currently there are efforts to find a less invasive diagnostic procedure. Niedoszytko et al. (2011) found significant differences in gene expression profile in ISM patients with insect venom anaphylaxis compared to ISM patients without anaphylaxis in history. Authors insist that further studies in larger group of patients are required to validate their results for the development of a predictive tool to be used in clinical practice.

The aim of the study was to analyse the gene expression differences in mastocytosis patients with and without food and drug hypersensitivity, and IVA.

## Materials and Methods

### Patients

A total of 57 Caucasian patients with mastocytosis, treated at the Department of Allergology, Medical University of Gdańsk were studied [median age 41.8; range 18–77 years; 15 (26.3 %) males and 42 (73.7 %) females]. All patients underwent standard diagnostic procedures in accordance with the WHO and ECNM (European Competence Network on Mastocytosis) standards, including bone marrow examinations with histopathological, cytological and flow cytometric (CD2, CD25) evaluation, KIT D816V mutation and basal serum tryptase level analyses.

The study was approved by the Ethical Commission of the Medical University of Gdańsk, Poland (No. NKEBN/151/2010). A written consent from an informed patient was obtained from the study participants.

### Collection of Blood Samples

Tempus Blood RNA Tubes (Applied Biosystems^®^, USA) were used for RNA sampling. All tubes were frozen and stored in −80 °C until RNA isolation (maximal period 6 months). RNA was isolated using the Tempus™ Spin RNA Isolation Kit (Ambion^®^, USA).

The quality and concentration of RNA were determined using NanoDrop and 2100 Bioanalyzer (Bioanalyzer, Bio-Rad, USA). Only samples with RNA integrity number >7.5 were used for further analysis. All RNA samples were stored in −80 °C until Reverse Transcription PCR (maximal period 12 months).

Reverse transcription was performed using the High Capacity cDNA Reverse Transcription Kits RNase Inhibitor (Invitrogen™, USA), which delivers extremely high-quality, single-stranded cDNA 0.02–2 µg total RNA. Reactions were scaled up to 100 µL to generate 10 µg of cDNA from a single reaction. The cDNA samples were stored in −20 °C until real-time RT-PCR (period 2–6 months).

### Gene Expression

Studied genes were chosen based on our previous study on the differences in gene expression among mastocytosis patients with an IVA (Niedoszytko et al. 2011).

Quantitative RT-PCRs of 11 genes plus ribosomal 18S RNA were run using TaqMan^®^ Array Micro Fluidic Cards (Applied Biosystems, USA) in 7900HT Fast Real-Time PCR system with preinstalled TaqMan^®^ Array Micro Fluidic Card Thermal Cycling Block, according to the manufacturer’s protocol.

One channel of a microfluidic card was loaded with a mix of 45 µl RNA-ase free water (Nuclease-Free Water (not DEPC-Treated) Ambion^®^, USA), 50 µL TaqMan^®^ Master Mix and 5 µl of cDNA to obtain 25 ng cDNA in the reaction mix. This cDNA template corresponded to 25 ng of total RNA.

### Data Analysis

Relative gene expression values were calculated using the ΔΔCt method (Schefe et al. 2006) using the Sequence Detection System 2.2.1 software (Applied Biosystems, USA). The ΔΔCt method gave the amount of the target gene normalized to an endogenous reference gene and relative to a calibrator sample (reference for all samples). The raw gene expression values were normalized according to the expression of ribosomal 18S RNA. The normalized expression of each gene was calibrated by its expression in a virtual calibration sample.

A statistical analysis was performed using Statistica v.10 StatSoft (Tulsa, USA). In the statistical analysis, the Mann–Whitney *U* test, Students *t* test, Wilcoxon test, Pearson correlation test and logistic regression analysis were used.

*p* values less than 0.05 were considered to be associated with statistical significance.

### Food and Drug Hypersensitivity and IVA Diagnosis and Treatment

Patients were asked for symptoms of anaphylactic reactions during a standard diagnosis and, additionally, a questionnaire focused on symptoms of insect, drug and food hypersensitivity was used. The skin prick test and the specific immunoglobulin E (sIgE) measurement where performed in cases where the immunological mechanism of an allergy was suspected. The food challenge was not used due to the high risk of an anaphylactic reaction in mastocytosis patients. IVA was diagnosed in accordance with European Academy of Allergy and Clinical Immunology (EAACI) guidelines and included specific IgE evaluation, and both skin and intracutaneous tests in all patients in concordance with the symptoms of IVA in medical history. The drug hypersensitivity (non-steroid anti-inflammatory drugs, antibiotics, local anesthetics) was diagnosed in accordance with EAACI/ENDA (European Network for Drug Allergy) guidelines and was confirmed by both skin prick and/or intracutaneous tests which were followed by the drug provocation tests (DPT) in selected group of patients. Before DPT, the evaluation of the individual risk–benefit ratio was estimated. DPTs were performed with all precaution measures in the inpatient clinic. DPTs were positive if they reproduced the original symptoms or objective symptoms of intolerance as urticaria or a drop of at least 20 % in FEV1 in spirometry.

Patients were treated according to the ECNM standards. In the event of food or drug hypersensitivity, avoidance of the culprit allergen was advised. Venom immunotherapy was initiated in ten patients (76.9 %) with confirmed IVA (*n* = 13). The other three patients (23.1 %) are due to start venom immunotherapy in the near future. The treatment was performed according to an ultrarush (wasp) or rush (bee) protocol μ with the maintenance dose of 100 μg. According to recommendations, this therapy should be performed lifelong in mastocytosis patients. Patients were trained in self-management of anaphylaxis, avoidance of hidden allergen sources and cross reacting allergens.

## Results

**Fig. 1:**
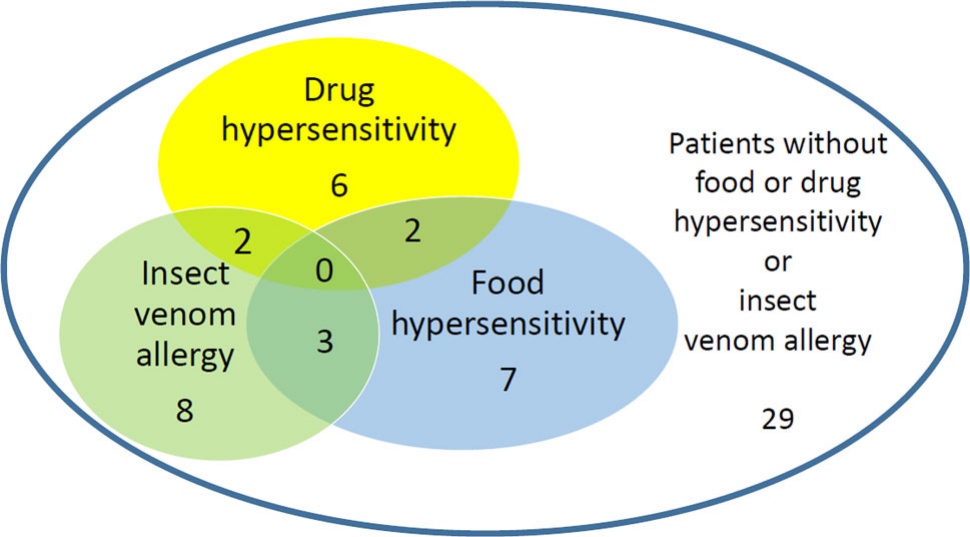
The prevalence of food and drug hypersensitivity and insect venom allergy in studied mastocytosis patients

The whole (*n* = 57) study group included 23 patients with cutaneous mastocytosis (CM; 40 %), 32 with ISM (56 %), 2 patients with smouldering systemic mastocytosis (4 %). The symptoms of food or drug hypersensitivity or IVA were found in 28 subjects (49 %), while 29 patients (51 %) were free of such symptoms (Fig. 1).

Symptoms of food hypersensitivity were found in 12 patients (21 %), including 3 patients (13 %) with CM, and 9 (28 %) with ISM. The factors responsible for the symptoms were (number of patients in brackets): fish (4), milk (3), chocolate (3), red wine/alcohol (3), apple (3), nuts (3), orange (2), banana (2), strawberries (2), meat (2). IgE-mediated food allergy was diagnosed in 3 ISM patients (5.2 %).

IVA was confirmed in 13 patients (22.8 %) including 6 patients (10.5 %) with CM and 7 patients (12.3 %) with ISM.

Drug hypersensitivity was diagnosed in ten patients (17.5 %) including six patients (10.5 %) and four patients (7 %) with ISM. Drugs causing allergic reactions included nonsteroidal anti-inflammatory drugs (*n* = 6), antibiotics (*n* = 2), local anesthetics (*n* = 3).

The prevalence of food and drug hypersensitivity and IVA is presented in Fig. 1. None of the patients presented all types of studied hypersensitivity and allergy. Food and drug hypersensitivity were found in two patients, IVA occurred in two patients with drug and two patients with food hypersensitivity.

No differences in tryptase level, KIT mutation, CD2 and CD 25 expression were observed according to the presence of food and drug intolerance, and IVA.

Significant differences in gene expression were found for TRAF4 (*p* = 0.008) in the comparison of the mastocytosis patients with and without concomitant food hypersensitivity (Table 1). The difference was significant in ISM (*p* = 0.04), not CM patients. It was also significant in the sub-group of patients with a food allergy (*p* = 0.04).

**Table 1:** Gene expression in patients with mastocytosis with food hypersensitivity compared to patients without symptoms of food hypersensitivity

Gen	*p* value
ABI3BP-Hs00227206_m1	0.91
ANKRD6-Hs00208902_m1	0.03
B3GAT1-Hs00218629_m1	0.09
CDC42BPA-Hs00177522_m1	0.24
DVL1-Hs00182896_m1	0.08
G0S2-Hs00274783_s1	0.17
HLA-DRB4-Hs03027795_uH	0.44
JUP-Hs00158408_m1	0.08
MAP2K3-Hs00177127_m1	0.13
TNFRSF4-Hs00533968_m1	0.09
TRAF4-Hs00188755_m1	0.008*

* Statistically significant difference

Moreover, significant differences in gene expression were found for B3GAT1 (*p* = 0.003) in patients with IVA compared to patients who did not react to an insect sting. B3GAT1 gene was underexpressed in patients with anaphylaxis (Table 2). There were no statistical significant differences in gene expression profiles depending on the history of drug hypersensitivity in the medical history.

**Table 2:** Gene expression in patients with IVA compared to patients without IVA

Gen	*p* value
ABI3BP-Hs00227206_m1	0.87
ANKRD6-Hs00208902_m1	0.64
B3GAT1-Hs00218629_m1	0.003*
CDC42BPA-Hs00177522_m1	0.86
DVL1-Hs00182896_m1	0.51
G0S2-Hs00274783_s1	0.51
HLA-DRB4-Hs03027795_uH	0.62
JUP-Hs00158408_m1	0.47
MAP2K3-Hs00177127_m1	0.07
TNFRSF4-Hs00533968_m1	0.24
TRAF4-Hs00188755_m1	0.50

* Statistically significant difference

## Discussion

The results of the study indicate the increased expression of TRAF4 in mastocytosis patients with food hypersensitivity and decreased expression of B3GAT1 in mastocytosis patients with IVA. There were no significant differences in gene expression in patients with mastocytosis depending on the occurrence of drug hypersensitivity. The symptoms of IVA were found in 12 (21 %) of subjects; food hypersensivity in 12 (21 %) of patients and drug hypersensitivity in 10 (17 %) of mastocytosis patients. Interestingly, the co-occurrence of studied food and drug hypersensitivity or IVA was found only in seven cases. It may be the reason for the differences in gene expression indicating the role of TRAF4 in food hypersensitivity and B3GAT1 in IVA as the patient groups and the pathophysiology of the reaction is different.

TRAF4 is crucial in developmental and morphogenic processes involved in oncogenesis and inflammation (Rousseau et al. 2011). Except the high differences in the expression of TRAF4 between control and neoplastic cells, the differences in inflammatory diseases like Crohn disease (Marinis et al. 2011; Rousseau et al. 2011) or airway inflammation (Zepp et al. 2015) were described. Recent studies indicate that TRAF4-mediated SMURF2-dependent degradation of DAZAP2 is a crucial step in interleukin (IL)-25 signalling. IL-25 is the only member of IL-17 cytokine family involved in Th2-associated cytokine production (IL-4, IL-5, IL-9 and IL-13), eosinophil recruitment, IgE production and innate immune response (Zepp et al. 2015). These mechanisms are common in allergic diseases including food allergy, thus, a higher TRAF4 expression may increase the risk of food hypersensitivity. Our data support the identification of the increased TRAF4 expression in airway inflammation (Zepp et al. 2015), Crohn disease (Marinis et al. 2011; Rousseau et al. 2011) and indicate the need for further studies.

B3GAT1 gene is localized on 11q25 chromosome and is a member of the glucuronyltransferase gene family. This gene product functions as the key enzyme in a glucuronyl transfer reaction during the biosynthesis of the carbohydrate epitope HNK-1, known also as CD57. It acts to add a glucuronic acid to the terminal *N*-acetyllactosamine (Lac) disaccharide to form the HNK-1 epitope precursor. CD57-positive T cells are important effectors of immune regulation and cytotoxicity. Olloquequi et al. (2011) found a significant and specific increase in the follicular density of CD57^+^ cells in patients with chronic obstructive pulmonary disease (COPD), which support the hypothesis of local immune dysfunction. Furthermore, this study proved that COPD patients exhibit a significant increase in the follicular density of CD57^+^ cells compared to nonsmokers and smokers without COPD. This finding shed light on the role that lymphoid folliculles could play in the disease, since there is an evidence pointing toward CD57 as a marker of lung inflammation (Palmer et al. 2005) or even as a marker of general immune dysfunction independent of the underlying disease (Focosi et al. 2010). Moreover, it has recently been demonstrated that CD57 antigen is also a marker of terminally differentiated cells with a high cytolytic potential (Chattopadhyay et al. 2009). It is considered that CD57 expression on CD8^+^ T cells, CD4^+^ T cells, and NK cells is a general marker of proliferative inability, a history of more cell divisions, and short telomeres (Brenchley et al. 2003). CD57 is involved in apoptosis and lack of proliferation. Moreover, the proliferative defects and apoptotic nature observed within HIV-specific CD8^+^ T cells can be predicted by expression of CD57 on cells. The presence of these cells, however, does not reflect a defect particular to the immune response to HIV or an effect limited to any particular virus, but simply reflects the normal consequence of persistent immune activation (Brenchley et al. 2003).

Our previous data indicated that genes involved in pathways responsible for the development of cancer may be related to the risk of an anaphylactic reaction to insect venom. We hypothesized that a more pronounced mast cells dysfunction in patients without a history of anaphylaxis is present (Niedoszytko et al. 2011). The current study is performed in a group of patients with less advanced mastocytosis, also including CM patients. Thus, the differences in gene expression responsible for a neoplastic transformation were not found. The observation of a higher expression of TRAF4 related to Th2 inflammation confirms another observation of the role of the IL-13 gene polymorphism in the pathogenesis of mastocytosis and frequent symptoms of food hypersensitivity affecting this group of patients (Nedoszytko et al. 2009). The genetics of anaphylaxis and intolerance are poorly understood. New observations describing the role of calcitonin gene-related peptide, the neurotransmitter of enteric sensory neurons responsible for the microtubule organization and interaction between mucosal mast cells and the development of food allergy, prove that food hypersensitivity mechanisms involve more pathways than the classic IgE response (Kim et al. 2014). The identification of novel targets like TRAF4 may constitute a research field focused on new therapeutic targets (Kim et al. 2014).

The opinions concerning food hypersensitivity in mastocytosis were conflicting. Studies on skin prick test and sIgE levels in mastocytosis patients do not show higher prevalence than in the general population (González de Olano et al. 2007). Similarly, the proportion of patients with an elevated tryptase level is higher in patients with an IVA than in subjects suffering from a food allergy (Bonadonna et al. 2009a, b). The studies based on the symptoms and broader mechanisms of food hypersensitivity, including histamine hypersensitivity (Brockow et al. 2008; Cifuentes et al. 2013; Koga et al. 2011; Prieto-García et al. 2015; Vlieg-Boerstra et al. 2005), present clinically important systemic reactions in this group of patients. Our results confirm that the prevalence of food hypersensitivity in mastocytosis patients (21 %) is higher than in the comparable age group of adults in Poland (5 %) (Samolinski 2015). The present study also adds a genetic trace in the pathology of the symptoms described as important by half of the patients (Jennings et al. 2014). The mechanism of hypersensitivity may be related to the TRAF4-mediated SMURF2-dependent degradation of DAZAP2 (Zepp et al. 2015), irrespective of the type of hypersensitivity (allergic or non-allergic). The variety of symptoms caused by food hypersensitivity and the treatment methods, including food avoidance, indicates the crucial role of a dietitian and patient education. In contrast, the IVA is mediated by IgE-dependent allergy thus the lifelong insect venom immunotherapy is crucial to prevent fatal reactions.

In conclusion, the TRAF4 expression was higher in mastocytosis patients with food hypersensitivity in their medical history. B3GAT1 gene and antigen CD57 play an important role in immune system and neoplasm processes. CD57 is a marker of well-differentiated cells with high cytotoxic potential. Moreover, CD57 is a marker of the immune system dysfunction regardless of the underlying disease. CD57 lymphocytes are involved in many chronic processes associated with the activation of immune system, such as viral infections, inflammatory disorders and also neoplasms, as well as physical stress and aging. The variation in the glycosylation of plasma proteins caused by the polymorphisms of B3GAT1 could be a predisposing or prognostic factor in numerous diseases.
